# Special Issue of *Materials* Focused on “Electrical, Thermal and Optical Properties of Nanocarbon Materials”

**DOI:** 10.3390/ma15051649

**Published:** 2022-02-22

**Authors:** Dawid Janas

**Affiliations:** Department of Organic Chemistry, Bioorganic Chemistry and Biotechnology, Silesian University of Technology, B. Krzywoustego 4, 44-100 Gliwice, Poland; dawid.janas@polsl.pl; Tel.: +48-32-237-10-82

Due to their extraordinary properties, nanocarbon materials such as carbon nanotubes (CNTs) or graphene have been at the forefront of research for the past few decades. In particular, the remarkable electrical, thermal, and optical properties have attracted considerable interest from the scientific community. Although a substantial amount of research conducted in the early days may have been overly optimistic, the field is currently past its hype peak, exploring the most feasible implementation routes. Having reached the plateau of productivity, more and more reports come out from this area, which displays how the aforementioned properties of carbon nanostructures can be exploited in real life.

This Special Issue aimed to gather reports on how the electrical, thermal, and optical characteristics of nanocarbon can be utilized. Overall, six contributions were published in this collection, which illustrates the application opportunities for these materials well.

The properties of carbon nanoarchitectures are somewhat sensitive to the conditions in which they are applied. Due to this relation, they can be used as sensors to monitor the external environment. Firstly, it was demonstrated that the surrounding gas atmosphere can greatly impact the electrical properties of CNT networks [1]. The electrical conductivity of CNT films was changed when the material was exposed to vacuum or a selection of gases such as methane, ethylene, oxygen, ammonia, nitrogen, argon, and hydrogen. In particular, the latter conditions notably influenced the electronic characteristics of the CNT ensemble. Simultaneously, it was shown that under non-oxidizing conditions, such CNT films can be electrically heated to very high temperatures. Secondly, Santos and co-workers reported that CNT networks can be used as strain sensors [2]. The authors studied the impact of CNT alignment, and it was concluded that the anisotropic films were much more sensitive to strain than isotropic materials. Most importantly, it was possible to infer the strain direction because of the fact that CNTs were ordered. Besides that, proper assembly of CNTs ensured appreciable mechanical properties of the network.

Furthermore, Taborowska and colleagues presented the merits of macroscopic CNT assemblies such as spun CNT films or fibers [3], the production of which does not involve CNT powders. The article gauged the implementation potential of such materials in a broad spectrum of electronic applications. The absence of binders in the synthesis process, which could contaminate the final material, and its axial alignment reveal that these CNT formulations can be highly useful in several areas of materials engineering. For example, spun CNT fibers are very conductive, and due to their yarn-like nature, they can be easily embedded in fabrics to create e-textiles.

The importance of orientation was also indicated in the contribution written by Li et al. [4], who deposited reduced graphene oxide onto aligned magnetic carbon fiber (MCF) skeleton. The reduced graphene oxide flakes on the surface of MCF provided appreciable values of thermal conductivity of the composite material, i.e., >600 W/m·K. As a result, the flexible and mechanically strong hybrid was used for heat dissipation.

Last but not least, rather than dissipate heat, CNTs can be employed to generate electrical energy from it. CNTs exhibit a Seebeck effect, so they can be exploited for thermoelectrics. Kumanek and co-workers showed that the capabilities of multi-walled CNTs for thermal energy recovery might be adjusted by simply changing the synthesis parameters [5]. Consequently, it is possible to optimize the microstructure and chemical composition of the material to enhance the electrical conductivity and Seebeck coefficient. When both of these values are high, heat can be efficiently converted into electricity. Moreover, the performance of single-walled CNTs in this field of exploitation can also be boosted by modification of the inherent electronic character of the material. Podlesny et al. reported that sorting of large-diameter single-walled CNTs into metallic and semiconducting fractions provides materials of markedly different thermoelectric capabilities [6]. This finding once again underlines the importance of CNT processing, which often predetermines whether the material will be suitable for a certain application or not.

In conclusion, the recent progress in the area of carbon nanostructures is encouraging as more and more published ideas every year reach high levels of technology readiness. The hope is that the presented collection of articles will serve as helpful guidance and inspiration to the newcomers to the field to progress it further.
